# Retraction: Infantile colic, facts and fiction

**DOI:** 10.1186/1824-7288-40-9

**Published:** 2014-03-11

**Authors:** Abdelmoneim EM Kheir

**Affiliations:** 1 Department of Paediatrics, Faculty of Medicine, University of Khartoum, Khartoum, Sudan

## Retraction

This article [1] has been retracted by the author due to extensive text overlap with a previous publication by Roberts *et al*. [2]. The author apologises for any inconvenience caused.
